# Exerkines During Pregnancy: Linking Maternal Exercise with Cardiometabolic Health—A Narrative Review

**DOI:** 10.3390/medsci14060630

**Published:** 2026-10-02

**Authors:** Iva Sklempe Kokic, Mateja Znika, Vesna Brumnic, Stjepan Jelica, Tomislav Kokic

**Affiliations:** 1 Faculty of Kinesiology Osijek, Josip Juraj Strossmayer University of Osijek, 31000 Osijek, Croatia; 2 Department of Physiotherapy, University North–University Center Varazdin, 42000 Varazdin, Croatia; mznika@unin.hr; 3 European Centre Maribor, Alma Mater Europaea University, 2000 Maribor, Slovenia; 4 Department of Health Studies, University of Applied Sciences “Lavoslav Ruzicka” in Vukovar, 32000 Vukovar, Croatia; vbrumnic@vevu.hr (V.B.); sjelica@vevu.hr (S.J.); 5 Orthopaedics and Trauma Department, General County Hospital Vinkovci, 32100 Vinkovci, Croatia; tkokic@gmail.com

**Keywords:** pregnancy, physical exercise, exerkines, gestational diabetes mellitus, hypertensive disorders of pregnancy, maternal obesity, irisin, placental function

## Abstract

Physical exercise during pregnancy is associated with important maternal and foetal health benefits. These include reduced risks of gestational diabetes mellitus (GDM), hypertensive disorders of pregnancy (HDP), and excessive gestational weight gain. However, the molecular mechanisms underlying these benefits remain incompletely understood. Exerkines are exercise-responsive signalling molecules released from skeletal muscle and other metabolically active tissues and may contribute to metabolic, vascular, inflammatory, and mitochondrial adaptations. This narrative review critically examines current evidence linking exerkines with maternal exercise and cardiometabolic adaptation during pregnancy, with particular emphasis on GDM, HDP, maternal obesity, placental function, and maternal–placental–foetal communication. Evidence was identified through a comprehensive, iterative search of PubMed/MEDLINE, Scopus, and Web of Science primarily covering literature published between January 2016 and July 2026. Priority was given to human pregnancy studies and clinically relevant outcomes. Pregnancy-specific evidence is currently strongest for irisin, although findings remain heterogeneous. Acute exercise studies also indicate transient changes in circulating fibroblast growth factor 21. Evidence for β-aminoisobutyric acid, apelin, adiponectin, chemerin, and interleukin-6 is more limited and frequently relies on experimental or non-pregnant populations. Overall, current evidence supports exerkines as plausible contributors to the cardiometabolic adaptations associated with maternal exercise, but does not establish them as causal mediators. Their principal value at present lies in providing a biological framework linking established clinical benefits of prenatal exercise with metabolic, vascular, inflammatory, and placental adaptations, while identifying specific pathways that require confirmation in pregnancy-specific longitudinal and intervention studies.

## 1. Introduction

Cardiometabolic health during pregnancy has become a major public health priority because of the prevalence and consequences of gestational diabetes mellitus (GDM), hypertensive disorders of pregnancy (HDP), and maternal obesity. The International Diabetes Federation estimated that hyperglycaemia in pregnancy affected approximately 23.0 million (19.7%) of live births to women in 2024, with GDM accounting for most cases [1]. Hypertensive disorders of pregnancy complicate approximately 5–10% of pregnancies worldwide and remain a major cause of maternal and foetal morbidity and mortality [2,3]. Obesity during pregnancy is associated with higher risks of GDM, gestational hypertension, preeclampsia, caesarean birth, and neonatal complications, as well as longer-term cardiometabolic consequences for mother and offspring [4,5].

Although GDM, HDP, and maternal obesity are clinically distinct, they share several pathophysiological features, including insulin resistance, chronic low-grade inflammation, oxidative stress, endothelial dysfunction, altered adipokine signalling, mitochondrial dysfunction, and placental maladaptation. These complications can therefore be viewed, at least partly, as different manifestations of impaired maternal cardiometabolic adaptation to pregnancy [4,6].

Regular physical activity is one of the few non-pharmacological strategies that can improve several of these outcomes simultaneously. International guidelines recommend that women with uncomplicated pregnancies accumulate at least 150 min of moderate-intensity physical activity each week [7,8,9]. Scientific evidence indicates that appropriately prescribed prenatal exercise reduces the risk of GDM and HDP, helps prevent excessive gestational weight gain, and improves cardiorespiratory fitness and psychological well-being without increasing the risk of adverse obstetric or neonatal outcomes [10,11].

Despite the well-established clinical benefits of prenatal exercise, the biological mechanisms responsible for these effects are not yet fully understood. Beyond increasing energy expenditure and improving physical fitness, exercise influences glucose regulation, vascular homeostasis, inflammatory signalling, oxidative balance, mitochondrial function, and placental physiology. Skeletal muscle is now recognised as an endocrine organ that communicates with other tissues through exercise-responsive signalling substances [12]. Collectively termed exerkines, these substances comprise peptides, proteins, metabolites, lipids, nucleic acids, and extracellular vesicles released from skeletal muscle as well as adipose tissue, the liver, vascular endothelium, bone, and other organs in response to acute or repeated exercise [13,14]. Experimental and translational studies indicate that exerkines influence insulin sensitivity, glucose uptake, endothelial function, angiogenesis, inflammatory signalling, oxidative balance, mitochondrial metabolism, and tissue remodelling [14,15].

Many of these pathways are also involved in GDM, HDP, maternal obesity, and placental dysfunction. Exerkines possibly contribute to the systemic adaptations associated with maternal exercise. Their causal role in human pregnancy, however, has not been established.

Pregnancy adds further complexity because exercise-responsive signals operate within the maternal–placental–foetal unit, and the extent to which exerkines contribute to communication between these compartments remains uncertain.

Among the exercise-responsive factors of greatest interest in pregnancy are irisin, fibroblast growth factor 21 (FGF21), interleukin-6 (IL-6), apelin, adiponectin, chemerin, and β-aminoisobutyric acid (BAIBA). Pregnancy-specific intervention evidence is available for only a small number of these substances, and much of the scientific literature derives from non-pregnant populations or experimental models [16,17].

Although previous reviews have summarised exercise during pregnancy, individual exerkines, placental adaptations, or developmental programming, few have integrated these topics within a clinically oriented framework linking maternal exercise with cardiometabolic adaptation across the maternal–placental–foetal unit [16,17,18]. Thus, an important gap remains between the well-established clinical benefits of prenatal exercise and the limited, heterogeneous evidence identifying the biological signals that may mediate these effects during pregnancy. In particular, pregnancy-specific evidence for individual exerkines remains fragmented, much of the mechanistic evidence is extrapolated from non-pregnant or experimental models, and direct exerkine-mediated signalling across the maternal–placental–foetal unit has not been established in humans.

The aim of this narrative review is to evaluate exerkines as potential biological mediators linking maternal physical activity with cardiometabolic adaptation during pregnancy. Particular attention is given to pathways relevant to GDM, gestational hypertension, preeclampsia, maternal obesity, placental function, and offspring development. The review distinguishes established clinical benefits and pregnancy-specific observations from mechanisms derived primarily from experimental or non-pregnant evidence and identifies key limitations and priorities for clinically relevant research.

## 2. Materials and Methods

This narrative review was developed through a comprehensive and iterative literature search of the PubMed/MEDLINE, Scopus, and Web of Science databases. The aim was to identify studies investigating exerkines during pregnancy and their potential role in maternal cardiometabolic adaptation. We considered primarily articles published between January 2016 and July 2026. Earlier landmark publications were incorporated when considered essential for providing historical context or explaining fundamental mechanisms. We performed the final literature search on 10 July 2026.

The literature search was performed using combinations of Medical Subject Headings (MeSH) terms and free-text keywords, including pregnancy, physical activity, exercise, exercise training, exerkines, myokines, adipokines, hepatokines, placenta, placental adaptation, maternal exercise, gestational diabetes mellitus, hypertensive disorders of pregnancy, gestational hypertension, preeclampsia, maternal obesity, cardiometabolic health, maternal–placental–foetal communication, angiogenesis, oxidative stress, inflammation, insulin resistance, irisin, fibroblast growth factor 21 (FGF21), interleukin-6 (IL-6), apelin, adiponectin, chemerin, β-aminoisobutyric acid (BAIBA), and extracellular vesicles. Boolean operators (AND/OR) were applied to combine search terms where appropriate. The search was conducted iteratively according to the topic being examined. Individual candidate exerkines and related terms were combined with pregnancy-related terms, with additional searches incorporating exercise or physical activity and relevant clinical, placental, and physiological outcomes. Representative combinations included “pregnancy AND irisin”, “pregnancy AND chemerin”, and corresponding searches for the other candidate exerkines, followed where relevant by combinations with “exercise” or “physical activity”. Additional targeted searches addressed GDM, HDP, maternal obesity, placental adaptation, inflammation, oxidative stress, angiogenesis, insulin resistance, and mitochondrial function.

Priority was given to peer-reviewed publications in English and to higher-level evidence, including international clinical guidelines, umbrella reviews, systematic reviews, meta-analyses, randomised controlled trials, and prospective cohort studies. Current recommendations from the World Health Organisation (WHO), the American College of Obstetricians and Gynecologists (ACOG), and the Canadian Society for Exercise Physiology (CSEP) were also considered. Human pregnancy studies were prioritised when evaluating pregnancy-specific associations and responses to exercise. Where pregnancy-specific evidence was limited or unavailable, experimental pregnancy studies and evidence from non-pregnant populations were considered to evaluate biological plausibility, with these sources interpreted separately from direct human pregnancy evidence. Additional relevant publications were identified by screening the reference lists of publications retrieved during the search. Publications were not prioritised when they focused primarily on exercise performance, general molecular or cellular mechanisms without a clear connection to pregnancy or maternal cardiometabolic adaptation, or outcomes outside the clinical and biological scope of this review.

Evidence in this field comes from exercise physiology, placental biology, and clinical obstetrics and varies considerably in study design and methodology. A narrative approach was therefore used to integrate findings across these different areas. The literature was selected according to its relevance to the clinical and biological questions addressed in the review rather than through a formal systematic-review screening process; consequently, formal record-screening and exclusion counts were not generated. Particular emphasis was placed on studies investigating shared biological mechanisms linking maternal exercise with GDM, HDP, maternal obesity, placental adaptation, and maternal–placental–foetal communication.

Instead of cataloguing individual biomarkers, this review integrates current evidence to evaluate how coordinated exerkine signalling may contribute to maternal cardiometabolic adaptation during pregnancy while highlighting important limitations of the available evidence. In this review, the term exerkine is used broadly to encompass exercise-responsive signalling substances released from skeletal muscle and other metabolically active tissues, including adipokines and hepatokines, as opposed to being restricted to muscle-derived myokines.

## 3. Maternal Exercise as a Non-Pharmacological Strategy for Improving Cardiometabolic Health During Pregnancy

### 3.1. Current Recommendations for Physical Activity During Pregnancy

Regular physical activity is recommended for most women with uncomplicated pregnancies. The WHO, ACOG, and the Canadian guidelines consistently support exercise as part of routine antenatal care [7,8,9].

These guidelines recommend at least 150 min of moderate-intensity physical activity per week, preferably distributed across at least three days. Aerobic activities such as brisk walking, cycling, swimming, and water-based exercise should be combined with resistance training involving the major muscle groups. Guidelines also recommend including pelvic floor muscle training and activities that support balance and mobility [8,9]. Adherence to current physical activity guidelines is sufficient to achieve health benefits [19]. The type and intensity of exercise should be tailored to the woman’s pre-pregnancy fitness level [8,9]. International Society for the Study of Hypertension in Pregnancy (ISSHP) specifically recommends exercise as a preventive strategy for gestational hypertension and preeclampsia in women without contraindications [20].

Exercise prescription should be adjusted to pre-pregnancy activity, maternal health, gestational age, symptoms, and obstetric risk. Individualization remains fundamental because maternal fitness, previous physical activity, obstetric history, medical comorbidities, and pregnancy complications differ substantially between women. Previously active women can usually continue exercising with appropriate modifications, but previously inactive women should increase activity gradually. It is also important to reduce sedentary time and encourage regular movement [7,9].

Medical and obstetric assessment is important when pregnancy complications are present. Some conditions, including severe preeclampsia, uncontrolled hypertension, ruptured membranes, active preterm labour, persistent bleeding later in pregnancy, severe foetal growth restriction, and clinically significant cardiovascular or respiratory disease, may limit or preclude moderate-to-vigorous exercise [8]. Exercise recommendations should therefore be tailored to the woman’s clinical condition and incorporated into routine antenatal care.

### 3.2. Evidence for Maternal Benefits

The maternal benefits of prenatal exercise are supported by randomised trials, systematic reviews, and meta-analyses. Appropriately prescribed activity improves metabolic, cardiovascular, musculoskeletal, and psychological health without increasing adverse maternal or foetal outcomes in uncomplicated pregnancies [10,11].

Exercise can help prevent several pregnancy-related disorders, including GDM, excessive gestational weight gain, HDP, urinary incontinence, foetal macrosomia, lumbopelvic pain, anxiety, and prenatal depression [19]. Regular moderate-intensity activity improves peripheral glucose uptake and insulin sensitivity and reduces GDM risk, with preventive effects generally strongest when programmes begin before conception or early in pregnancy [10]. Exercise also contributes to glycaemic management after GDM has developed, although the magnitude of benefit varies according to intervention timing, exercise dose, adherence, baseline metabolic risk, and participant characteristics [21,22].

Prenatal exercise is associated with a lower risk of gestational hypertension, but the evidence for a reduction in preeclampsia is less consistent [23]. Improved endothelial function, vascular responsiveness, blood pressure regulation, and inflammatory control may contribute to these outcomes, although the clinical evidence for reduced HDP is stronger than the pregnancy-specific evidence for the proposed molecular mediators [10,11].

Regular activity limits excessive gestational weight gain and may attenuate some obesity-related pregnancy risks [24,25]. It should not be described as reducing maternal obesity during pregnancy. Exercise can improve metabolic health and reduce excessive weight gain even when maternal body mass changes little [4,10].

Exercise additionally supports cardiorespiratory fitness, functional capacity, and psychological well-being [8]. Evidence indicates reductions in depressive and anxiety symptoms and improvements in sleep and quality of life, reinforcing its value as a broad component of antenatal care [11,26,27]. These established clinical benefits provide the basis for examining possible biological mediators, including exerkines.

### 3.3. Evidence for Foetal and Placental Benefits

Maternal physical activity is compatible with healthy foetal growth and neonatal outcomes. In uncomplicated pregnancies, regular moderate-intensity exercise is not associated with increased risks of miscarriage, preterm birth, low birth weight, or small-for-gestational-age birth, and it reduces excessive foetal growth and macrosomia in metabolically high-risk pregnancies [10,11,19].

The placenta is a likely intermediary because it adapts to maternal metabolic and haemodynamic conditions. Human studies have reported associations between maternal physical activity and changes in placental morphology as well as selected angiogenic markers, whereas experimental studies suggest additional effects on vascularization, nutrient sensing, and cellular stress responses. However, given the heterogeneity in these findings, it cannot be concluded that exercise consistently improves all aspects of placental structure or mitochondrial function [28,29,30,31].

Maternal exercise may also influence developmental programming. Experimental models generally report favourable effects on offspring glucose regulation, body composition, and cardiovascular function. Human evidence is more limited and supports possible, long-term benefits for offspring cardiometabolic health [32].

Taken together, the clinical evidence indicates that maternal exercise benefits the mother without compromising foetal development and may modify placental function. Detailed placental morphology, angiogenic signalling, nutrient transport, and maternal–placental–foetal communication are considered in Section 5.

### 3.4. Current Knowledge Gaps: From Clinical Evidence to Molecular Mechanisms

Clinical evidence that prenatal exercise improves pregnancy outcomes is considerably stronger than evidence identifying the molecular mediators of these effects. Most intervention studies assess GDM, blood pressure, gestational weight gain, or birth outcomes without measuring exercise-responsive signalling molecules at the same time.

Pregnancy also alters endocrine, metabolic, inflammatory, and placental signalling across gestation. Cross-sectional measurements of individual biomarkers therefore provide limited information about tissue origin, timing, or causality. Longitudinal studies that combine standardised exercise interventions with repeated biological sampling and clinically meaningful outcomes are still uncommon [16,17].

Exerkines provide a useful framework for exploring how exercise may influence several physiological systems at the same time. In the following section, pregnancy-specific findings are considered separately from mechanisms identified mainly in experimental studies and non-pregnant populations.

## 4. Exerkines as Biological Mediators of Maternal Cardiometabolic Adaptation

Regular physical exercise induces coordinated metabolic, vascular, inflammatory, and mitochondrial adaptations that extend beyond the contracting skeletal muscle. These responses are accompanied by the release of exercise-responsive signalling molecules from skeletal muscle, adipose tissue, the liver, vascular endothelium, bone, and other metabolically active organs [14,15]. These signals act within a changing endocrine environment and interact with the placenta, a transient organ that regulates maternal and foetal physiology.

Exerkines should therefore be considered candidate contributors to maternal adaptation, not established causal mediators. The evidence below is organised by physiological function and differentiates pregnancy-specific observations from mechanisms demonstrated mainly in experimental or non-pregnant settings.

### 4.1. Exerkines Regulating Glucose Homeostasis and Insulin Sensitivity

Maintenance of glucose homeostasis is fundamental to a healthy pregnancy. As gestation progresses, physiological insulin resistance develops to ensure an adequate nutrient supply for the growing foetus. This adaptation is essential for normal foetal development. However, excessive insulin resistance may exceed maternal pancreatic compensatory capacity, contributing to GDM. Gestational diabetes mellitus is associated with increased risks of hypertensive disorders of pregnancy, foetal overgrowth, operative delivery, and long-term cardiometabolic disease in both mother and offspring. Regular physical activity is among the most effective non-pharmacological interventions for improving insulin sensitivity and reducing the incidence of GDM [21,22,23].

While the clinical benefits of maternal exercise are well established, the biological mechanisms responsible for these improvements are still being elucidated. Exercise stimulates the release of numerous bioactive molecules, collectively referred to as exerkines, from skeletal muscle, adipose tissue, the liver, and other metabolically active organs. These molecules do not appear to act independently. Their effects involve several interconnected pathways related to glucose uptake, insulin signalling, mitochondrial metabolism, and whole-body energy homeostasis.

#### 4.1.1. Irisin

Among the currently identified exerkines, irisin has received the greatest attention in pregnancy research. Irisin is generated through cleavage of the transmembrane fibronectin type III domain-containing protein 5 (FNDC5) following activation of the peroxisome proliferator-activated receptor gamma coactivator 1-alpha (PGC-1α) pathway during exercise [33]. Experimental studies have shown that irisin enhances glucose uptake by promoting glucose transporter type 4 (GLUT4) translocation and activating adenosine monophosphate-activated protein kinase (AMPK), thereby improving insulin sensitivity in skeletal muscle and adipose tissue [34,35]. Additional evidence suggests that irisin may preserve mitochondrial function and attenuate oxidative stress, both of which are relevant to pregnancies complicated by obesity or GDM [36].

Human pregnancy studies provide encouraging but inconsistent evidence. Women with decreased plasma levels of irisin during the first trimester of pregnancy have an increased risk of GDM [37] although the clinical utility of circulating irisin for early identification of GDM risk has not been established. Higher circulating irisin concentrations after delivery in women who had experienced GDM compared with controls were reported by Ebert et al. [38], although concentrations did not differ significantly between groups during pregnancy. Several investigations have reported lower circulating irisin concentrations in women with GDM than in uncomplicated pregnancies, whereas others observed no significant differences or even higher concentrations [39,40]. These discrepancies probably reflect differences in gestational age, maternal adiposity, disease severity, treatment, sample handling, and analytical methodology.

Irisin levels were lower in less physically active pregnant women but increased in women who performed aerobic exercise three times per week for eight weeks. These increases were negatively correlated with fasting glucose, insulin concentrations, and glycated haemoglobin [41]. This suggests that prenatal exercise may improve glucose homeostasis markers in healthy women and compensate for metabolic changes induced by pregnancy [41]. Active pregnant women experience a post-exercise increase in irisin which is also related to a more favourable lipid profile [42]. Consequently, irisin should currently be regarded as a promising candidate involved in metabolic adaptation. Regular exercise during pregnancy may increase irisin levels, while experimental evidence suggests that irisin can promote white adipose tissue beiging/browning, improve glucose homeostasis, and enhance energy expenditure, which may represent one of the mechanisms through which exercise contributes to the prevention of GDM [43].

#### 4.1.2. Fibroblast Growth Factor 21

Fibroblast growth factor 21 is an endocrine regulator involved in glucose and lipid metabolism, insulin sensitivity, and metabolic flexibility [44]. Exercise-induced increases in circulating FGF21 have been reported in non-pregnant populations, where the molecule appears to promote glucose utilisation, fatty acid oxidation, and adaptive responses to metabolic stress. Experimental evidence also suggests interactions between FGF21, adiponectin, and AMPK-dependent signalling pathways that may contribute to improved insulin sensitivity [14,45].

Evidence during pregnancy remains scarce. One acute exercise study demonstrated that moderate-intensity walking increased circulating FGF21 concentrations together with erythropoietin and interleukin-15 in pregnant women, indicating that pregnancy does not abolish the endocrine response to exercise [46]. Currently, it is unknown if these transient changes contribute to sustained improvements in insulin sensitivity or reduced GDM risk.

Although altered circulating FGF21 concentrations have been described in women with GDM and obesity, it is uncertain if these changes represent compensatory metabolic responses or contribute directly to disease pathophysiology [47,48,49,50]. Circulating FGF21 concentrations may not directly reflect its biological activity. In non-pregnant populations and experimental models, acute exercise increases circulating FGF21, while chronic exercise may enhance tissue sensitivity to FGF21 despite lower circulating concentrations [15]. This distinction may be particularly relevant when interpreting altered FGF21 concentrations in metabolically complicated pregnancies. FGF21 may therefore represent a potential exercise-responsive mediator of maternal metabolic adaptation; however, pregnancy-specific exercise evidence remains limited, and altered circulating concentrations alone do not establish either a mechanistic role or clinical utility. Current evidence does not support its use as a biomarker or therapeutic target during pregnancy.

#### 4.1.3. β-Aminoisobutyric Acid

β-Aminoisobutyric acid is an exercise-responsive metabolite linked to PGC-1α activation during skeletal muscle contraction [51]. Evidence from experimental studies conducted outside pregnancy suggests that BAIBA promotes fatty acid oxidation, enhances metabolic health, and improves insulin sensitivity, primarily through activation of AMPK-related pathways [14,51,52]. These actions indicate a potential role in maintaining metabolic homeostasis during periods of increased energy demand.

To date, evidence regarding BAIBA in pregnancy is extremely limited, and no longitudinal human studies have examined its response to structured prenatal exercise. Its proposed role in maternal metabolic adaptation therefore remains speculative and requires confirmation in well-designed pregnancy-specific intervention studies. Accordingly, the comparatively limited discussion of BAIBA in this review reflects the scarcity of pregnancy-specific evidence rather than evidence of lesser biological importance.

#### 4.1.4. Apelin

Apelin is a peptide expressed in several maternal tissues, including skeletal muscle, adipose tissue, vascular endothelium, and the placenta. It binds to the apelin receptor (APJ), activating Gαi- and Gαq-dependent signalling pathways and increasing muscle metabolism [17]. Experimental evidence indicates that apelin contributes to glucose homeostasis by improving insulin sensitivity, facilitating glucose uptake, and supporting endothelial function. Apelin activates AMPK through Gαq to stimulate GLUT4 in fat and muscle [53,54,55]. Furthermore, physical exercise activates AMPK, which contributes to mitochondrial homeostasis and skeletal muscle metabolism and may improve insulin resistance and other metabolic disturbances [54].

In addition, apelin has been implicated in placental angiogenesis and metabolic regulation, providing biological plausibility for a role in maternal and placental adaptation to exercise; however, this role has not been demonstrated directly in human pregnancy [56].

Observational studies suggest associations between altered apelin concentrations and metabolic complications such as GDM and maternal obesity [57], but direct evidence that exercise-induced changes in apelin improve pregnancy outcomes is currently unavailable.

#### 4.1.5. Adiponectin

Adiponectin is secreted predominantly by maternal adipose tissue and has three receptors: adiponectin receptor 1 (AdipoR1), adiponectin receptor 2 (AdipoR2), and T-cadherin [28]. Adiponectin receptors are expressed in placental tissue, allowing maternal adiponectin to influence placental signalling and nutrient transport. Binding of adiponectin to AdipoR1/R2 activates several signalling pathways, including AMPK, p38 mitogen-activated protein kinase (MAPK), peroxisome proliferator-activated receptor-α (PPAR-α), Ras-associated protein Rab5, phosphatidylinositol 3-kinase (PI3K), and protein kinase B (AKT), contributing to its insulin-sensitising actions [58].

The effects of adiponectin appear to be tissue-specific during pregnancy. While adiponectin enhances insulin sensitivity in maternal metabolic tissues, experimental evidence indicates that it can inhibit placental insulin and mechanistic target of rapamycin (mTOR) signalling, thereby reducing placental nutrient transport and limiting foetal growth [59].

Low levels of adiponectin are associated with diabetes and cardiovascular disease [60]. Also, low levels of adiponectin during the first trimester of pregnancy are associated with a higher risk of GDM in obese mothers [61]. A recent systematic review further supports this association, showing that reduced circulating adiponectin concentrations are consistently associated with impaired glucose metabolism and insulin resistance in women with GDM [62]. There is also a negative relationship between adiponectin and inflammatory markers in metabolic syndrome as well as a negative correlation between adiponectin levels and obesity [28,63].

Outside pregnancy, aerobic exercise has been associated with higher adiponectin concentrations in adults with prediabetes and diabetes [64]. Adiponectin increases fatty acid oxidation and glucose uptake in skeletal muscle and decreases hepatic glucose production, thereby improving insulin sensitivity and glucose homeostasis [65]. Whether comparable exercise-induced changes in adiponectin contribute to improved glucose regulation during pregnancy remains uncertain.

#### 4.1.6. Chemerin

Chemerin also has a role in the regulation of obesity and metabolism. Levels of chemerin are increased in umbilical cord blood, peripheral blood, adipose tissue and placental tissue during GDM [66]. It also positively correlates with fasting blood sugar, 2 h postprandial glucose, fasting insulin, insulin resistance index, C-reactive protein, and insulin dose in GDM [67]. Chemerin regulates adipogenesis and fat metabolism [68]. Obese women and women with GDM have higher levels of serum chemerin than pregnant women with normal weight and without GDM [69,70]. Such increases in chemerin in GDM possibly contribute to insulin resistance in obese women with GDM [71].

Outside pregnancy, aerobic training combined with dietary intervention has been associated with lower chemerin concentrations in women with obesity [72]. Altered chemerin signalling has been implicated in the pathophysiology of GDM, although findings regarding circulating concentrations remain heterogeneous. However, evidence that prenatal exercise modifies chemerin or that such changes contribute to improved pregnancy outcomes is currently insufficient. Longitudinal pregnancy-specific studies are needed to distinguish whether chemerin primarily reflects maternal metabolic and inflammatory status or participates directly in exercise-related adaptation.

#### 4.1.7. Clinical Perspective

Current clinical evidence consistently demonstrates that regular physical activity improves maternal glucose regulation and reduces the risk of GDM. Exerkines provide a biologically plausible explanation for these benefits by influencing insulin sensitivity, glucose uptake, substrate utilisation, and cellular energy metabolism. Irisin currently has the strongest pregnancy-specific evidence, but findings remain inconsistent. For FGF21, BAIBA, apelin, adiponectin, and chemerin, much of the available evidence still comes from experimental studies or research in non-pregnant populations. The overall evidence is therefore heterogeneous in both quantity and quality. Pregnancy-specific studies are generally limited by small samples, differences in gestational timing and exercise exposure, and predominantly observational or cross-sectional designs, while differences in sampling and analytical methods may further contribute to inconsistent circulating exerkine concentrations. Consequently, current findings support biological plausibility but do not establish that individual exerkines mediate the clinical benefits of prenatal exercise. Their physiological roles, responsiveness to exercise, and potential clinical utility require confirmation in adequately powered longitudinal and intervention studies.

### 4.2. Exerkines Regulating Endothelial Function and Vascular Adaptation

Hypertensive disorders of pregnancy, including gestational hypertension and preeclampsia, are characterised by impaired cardiovascular adaptation and endothelial dysfunction [73]. Normal pregnancy involves cardiovascular adaptations, including systemic vasodilation, expansion of plasma volume, increased cardiac output, and remodelling of the uteroplacental circulation, together with increased nitric oxide availability. Failure of these adaptations contributes to placental hypoperfusion, oxidative stress, and systemic vascular dysfunction [3,6].

Regular prenatal exercise is associated with a reduced incidence of HDP while improving vascular responsiveness and blood pressure regulation [10,11,23]. Several exerkines discussed above in relation to glucose homeostasis, particularly apelin, irisin, and adiponectin, also have biological actions relevant to endothelial function and angiogenic signalling [17]. They are considered here specifically in relation to vascular adaptation and HDP. These mechanisms provide possible pathways through which exercise could contribute to improved vascular adaptation. However, direct mediation has not been demonstrated in pregnancy.

As noted above, apelin is expressed in both maternal and placental tissues. Experimental studies show that apelin-APJ signalling can activate PI3K/AKT and endothelial nitric oxide synthase pathways and support angiogenic responses [56]. Furthermore, animal studies suggest that maternal exercise influences apelin signalling and that apelin may contribute to exercise-induced improvements in placental vascular and metabolic function [29,74]. However, corresponding evidence demonstrating exercise-induced placental apelin secretion in human pregnancy is currently lacking. Its placental expression makes apelin a potential link between maternal metabolism, vascular adaptation, and placental function. Human studies have not yet shown that exercise-induced changes in apelin mediate a reduction in preeclampsia or gestational hypertension [56].

Acute exercise also produces a transient rise in IL-6, with contracting skeletal muscle representing an important source of exercise-induced IL-6. During pregnancy, however, IL-6 is also produced within gestational tissues, including the decidua and placenta, and circulating concentrations cannot be attributed exclusively to skeletal muscle [75]. This physiological response differs fundamentally from the chronic low-grade elevation of IL-6 observed in obesity and pregnancy complications. Rather than promoting sustained inflammation, exercise-induced IL-6 appears to contribute to glucose regulation and anti-inflammatory signalling through subsequent increases in interleukin-10 (IL-10) and interleukin-1 (IL-1) receptor antagonist and suppression of tumour necrosis factor-α (TNF-α) production [76]. IL-6 also has pregnancy-specific regulatory functions at the maternal–foetal interface and participates in the inflammatory signalling associated with parturition [75]. Thus, the biological significance of circulating IL-6 during pregnancy depends on its tissue source, timing, and physiological context. The broader role of IL-6 in inflammatory regulation is discussed in Section 4.3.

Irisin levels negatively correlate with systolic and diastolic blood pressure in women with preeclampsia who have decreased levels of serum irisin unrelated to body mass index or gestational age [77,78]. Recent experimental evidence further suggests that irisin may support trophoblast migration, invasion, proliferation, and vascular remodelling through signal transducer and activator of transcription 4/glucose transporter type 3 (STAT4/GLUT3) signalling. This provides a potential placental mechanism relevant to preeclampsia [79]. The antioxidant and anti-inflammatory effects of irisin have also been reported to attenuate endothelial dysfunction in an experimental rat model of preeclampsia [80]. On the other hand, Szumilewicz et al. [81] reported that in physically active women irisin did not have a significant role in blood pressure regulation during pregnancy.

Adiponectin has insulin-sensitising, anti-inflammatory, and endothelial-protective properties. Levels of adiponectin are found to be decreased in obese women with severe preeclampsia, but increased in normal-weight women with preeclampsia [82]. Evidence outside pregnancy indicates that exercise-induced changes in adiponectin are variable and may depend on metabolic status and the type, intensity, and duration of exercise [83]. These changes have not been adequately investigated in women with preeclampsia.

Chemerin is also associated with maternal adiposity, inflammation, and vascular dysfunction. Available studies support its role mainly as a marker of metabolic status rather than a primary mediator of exercise adaptation [17]. Circulating chemerin levels are significantly increased in women with preeclampsia compared with healthy pregnant women [68]. Chemerin levels are positively correlated with the ratio of soluble Fms-like tyrosine kinase-1 (sFlt-1) to placental growth factor (PlGF), a biomarker of preeclampsia severity [84]. Up-regulation of placental chemerin synthesis disturbs normal placental development via its chemokine-like receptor 1 (CMKLR1), contributing to foetal growth restriction and the development of preeclampsia [84]. In contrast, regular strength and endurance training decreases circulating chemerin concentrations in individuals with overweight or obesity [85]. It is not yet known if a comparable response occurs in pregnant women with obesity.

Overall, available evidence suggests that exerkines may contribute to vascular adaptation through effects on nitric oxide bioavailability, angiogenic signalling, inflammation, and oxidative balance. However, direct pregnancy-specific evidence linking exercise-induced exerkine responses with reduced HDP remains limited.

### 4.3. Exerkines Regulating Inflammation and Oxidative Stress

Low-grade inflammation and oxidative stress are common features of GDM, HDP, maternal obesity, and placental dysfunction. Physiological pregnancy includes changing inflammatory activity and moderate reactive oxygen species (ROS) production. Persistent inflammatory activation and excessive oxidative stress can impair insulin signalling, endothelial function, trophoblast biology, and placental perfusion [6,86].

IL-6 illustrates the importance of physiological context, as its metabolic and inflammatory effects depend on its tissue origin, magnitude and duration of elevation, and signalling pathway [15]. Classical IL-6 signalling through membrane-bound interleukin-6 receptor (IL-6R) is generally associated with regenerative and anti-inflammatory actions, while trans-signalling through soluble IL-6R is more closely linked to pro-inflammatory responses [87].

Chronic elevation is associated with obesity and inflammatory disease, whereas the temporary increase produced by contracting skeletal muscle during exercise is linked to glucose utilisation and subsequent anti-inflammatory responses, including increased IL-10 and IL-1 receptor antagonist and suppression of TNF-α production [76,88]. Currently, it is unknown if this physiological IL-6 response contributes directly to improved obstetric outcomes because pregnancy-specific intervention studies are limited.

Pregnancy-specific evidence suggests that the relationship between physical activity and inflammatory myokines may vary across gestation. In their scoping review, Goudreau et al. [16] found preliminary evidence of trimester-specific associations between maternal physical activity and circulating IL-6, IL-10, and TNF-α. While findings were inconsistent in early and mid-pregnancy, higher physical activity was more consistently associated with lower TNF-α concentrations in late pregnancy. This suggests that exercise-related myokine responses may depend on the changing inflammatory profile of pregnancy [16].

Adiponectin links metabolic and vascular regulation. It activates AMPK-dependent pathways, supports insulin sensitivity and endothelial nitric oxide production, and can suppress inflammatory signalling [89]. Lower adiponectin concentrations are frequently reported in obesity, GDM, and preeclampsia. Prenatal exercise studies have not shown a uniform response. Chemerin is associated with adiposity and inflammatory status, with limited evidence that exercise-related changes in chemerin directly improve pregnancy outcomes [17].

Irisin and FGF21 also have anti-inflammatory and antioxidant effects in experimental studies. These actions may involve improved mitochondrial function and cellular stress responses, but most of the evidence comes from non-pregnant models [15,90]. These findings therefore provide mechanistic plausibility rather than direct evidence of equivalent effects during human pregnancy.

Repeated exercise can strengthen endogenous antioxidant defence despite the temporary increase in ROS produced by an acute bout. Improved redox regulation may support maternal metabolism, vascular function, and placental health. Still, there are no direct studies combining prenatal exercise, exerkine measurement, and placental oxidative markers [91].

Inflammatory and oxidative pathways connect the metabolic, vascular, and placental effects discussed throughout this review. Exerkines possibly modulate these pathways, but the available pregnancy-specific evidence is insufficient to assign causality to individual molecules.

### 4.4. Exerkines Regulating Mitochondrial Metabolism

Mitochondrial dysfunction contributes to insulin resistance, endothelial dysfunction, oxidative stress, and placental disease [92,93]. Pregnancy substantially increases maternal and placental energy requirements. This necessitates coordinated adaptations in substrate utilisation, oxidative phosphorylation, and redox regulation. If these adaptive responses fail, this may contribute to GDM, HDP, and obesity-related placental dysfunction.

Regular exercise promotes mitochondrial biogenesis, respiratory capacity, lipid oxidation, and endogenous antioxidant defence. PGC-1α represents a central intracellular regulator of these adaptations. It also influences the expression of several exercise-responsive exerkines, including irisin and BAIBA. Irisin, FGF21, BAIBA, and apelin have each been linked to mitochondrial or substrate-metabolism pathways in experimental research. However, their specific contribution during human pregnancy is not certain [33,51].

The placenta also depends on mitochondrial function for nutrient transport, hormone synthesis, and cellular turnover [28]. Experimental studies suggest that maternal exercise improves placental metabolic signalling and reduces obesity-related dysfunction. On the other hand, direct human measurements of placental respiration after exercise interventions are very limited [28,29].

Mitochondrial adaptation may therefore provide a link between improvements in glucose regulation, vascular function, inflammatory control, and placental physiology. Evidence for this connection, however, comes largely from experimental models and non-pregnant populations. There are relatively few studies conducted during pregnancy. Direct evidence demonstrating that circulating exerkines are the principal mediators of mitochondrial adaptation during human pregnancy does not yet exist.

## 5. Maternal–Placental–Foetal Communication: Implications for Placental Function and Offspring Health

### 5.1. The Placenta as an Endocrine and Metabolic Interface

The placenta is the principal interface between maternal and foetal physiology. In addition to nutrient and gas exchange, it has endocrine, metabolic, vascular, and immunological functions that influence maternal adaptation and foetal growth [59,94,95].

Placental development requires trophoblast differentiation, spiral artery remodelling, angiogenesis, controlled inflammatory activity, and adequate cellular metabolism. Disturbances in these processes are implicated to varying degrees in HDP, foetal growth restriction, GDM, and obesity-related pregnancy complications [73,96].

Maternal physical activity may modify placental structure and function. Human studies have reported differences in placental exchange surfaces, villous vascular volume, cell proliferation, and selected angiogenic proteins among active women, while recent data show that placental structural associations with activity and sedentary behaviour are complex and may depend on maternal metabolic status [30,31,91,97,98,99,100].

The placenta also participates in communication between maternal tissues and the foetus through hormones, cytokines, growth factors, and extracellular vesicles. Several candidate exerkines have receptors or biological actions in placental tissue, but it is not yet known whether exercise-related changes in these molecules are responsible for the placental adaptations observed in humans [17,56]. An additional interpretive challenge is that circulating concentrations measured during pregnancy do not necessarily identify the tissue of origin. Exercise-responsive molecules may arise from several maternal tissues and, for some candidates, from the placenta itself. For example, FNDC5, the precursor of irisin, is expressed in human placental tissue [101], while apelin and its receptor APJ are also expressed in placental tissue [56]. Consequently, changes in maternal serum concentrations cannot automatically be attributed to skeletal muscle release or interpreted as direct evidence of an exercise-induced myokine response.

Maternal exercise therefore has documented clinical and placental associations, but the involvement of exerkines in maternal-placental signalling remains largely hypothetical. Current placental findings should be interpreted without assuming that they result from a single molecular pathway.

### 5.2. Effects of Maternal Exercise on Placental Adaptation

The placenta is highly responsive to maternal metabolic and vascular conditions. Evidence from human studies suggests that maternal physical activity may influence placental morphology and selected angiogenic pathways, although findings vary by population, activity assessment, and maternal metabolic status. Experimental models additionally suggest effects on nutrient sensing, metabolic signalling, and cellular stress responses, but these findings should not be assumed to represent equivalent responses in human pregnancy.

Early human studies reported exercise-related differences in placental volumetric composition and exchange surface areas [98]. Women who continued running throughout pregnancy also had greater placental villous vascular volume and cell proliferation at term than less active comparison groups [99]. These observational findings suggest structural adaptation but do not establish that all placental changes are clinically beneficial.

More recent human studies have examined placental structural and angiogenic adaptations to maternal physical activity. Regularly active pregnant women have also been reported to exhibit a more pro-angiogenic circulating profile, characterised by higher PlGF concentrations alongside lower sFlt-1 and soluble endoglin (sEng) concentrations [102]. Physically active women had higher placental vascular endothelial growth factor (VEGF) and vascular endothelial growth factor receptor-1 (VEGFR-1) expression in one cohort, while a prenatal exercise intervention increased placental angiogenin without evidence of greater oxidative or endoplasmic reticulum stress [91,100]. Still, findings are not consistent across studies. In women with obesity participating in the DALI study, higher moderate-to-vigorous physical activity was associated with greater villous density, but not with vessel area or angiogenic gene expression [30]. More recently, objectively measured moderate-to-vigorous physical activity in the FitMum study was associated with greater placental weight but not with villous density, vessel area, or placental expression of vascular growth factors [31]. These findings suggest that maternal physical activity may influence placental development, but do not support a uniform effect on placental vascularization or angiogenic signalling.

Placental nutrient transfer depends on maternal substrate availability, blood flow, trophoblast metabolism, transporters, and nutrient-sensing pathways. Prenatal activity and diet composition have been associated with expression of nutrient transporters and mTOR-related proteins, but the clinical meaning of these differences is not certain [103]. In contrast to the human findings described above, evidence for several proposed intracellular mechanisms derives primarily from experimental models. In obese pregnant mice, exercise restored placental vascular density and angiogenic signalling, altered AMPK and insulin-related pathways, and prevented foetal overgrowth [29]. This supports a possible mechanism, but it cannot be assumed that the same response occurs in human pregnancy.

Human evidence for improved placental mitochondrial respiration is less developed than evidence for morphology and selected angiogenic markers. Moderate prenatal exercise has not been shown to increase placental oxidative injury, and experimental studies support improved metabolic resilience, but direct measurements of human placental mitochondrial function remain uncommon [28,91,104].

Candidate exerkines such as apelin, irisin, FGF21, adiponectin, and IL-6 could influence placental vascular, metabolic, and inflammatory pathways. At present, however, human studies rarely combine standardised exercise interventions, repeated exerkine measurements, and direct placental analysis. As discussed above, circulating measurements alone cannot establish the tissue of origin of these molecules. These molecules should therefore be described as possible contributors, not proven intermediaries.

### 5.3. Maternal Exercise, Developmental Programming, and Offspring Health

The Developmental Origins of Health and Disease (DOHaD) framework proposes that the intrauterine environment influences later susceptibility to cardiometabolic disease. Maternal metabolic health and placental function are relevant not only to pregnancy outcomes but also to offspring development [59,105,106].

Prenatal exercise reduces GDM, HDP, and excessive gestational weight gain and may attenuate some risks associated with maternal obesity without increasing foetal growth restriction [10]. These maternal effects may create a more favourable intrauterine environment. However, the evidence for long-term offspring benefit is much stronger in animal models than in humans.

Experimental studies report improvements in offspring glucose regulation, body composition, skeletal muscle metabolism, and cardiovascular function after maternal exercise. Human studies are fewer, use heterogeneous exercise exposures, and generally have shorter follow-up. Experimental evidence indicates possible developmental programming effects rather than established prevention of later cardiometabolic disease [32,107]. Maternal exercise decreases the risk of macrosomia and stimulates a better control of glucose metabolism [108]. Maternal aerobic exercise has also been associated with improved infant neuromotor development [109]. Offspring born to exercising mothers have reduced risk of childhood obesity [110]. Additionally, some human studies have reported associations between maternal exercise and foetal cardiac autonomic measures, but these outcomes do not establish long-term cardiometabolic protection [111,112].

Potential mechanisms include improved maternal insulin sensitivity, reduced inflammation and oxidative stress, altered placental nutrient handling, and epigenetic regulation [32,107,113]. Exerkines may participate in these processes indirectly through maternal or placental effects. However, it is not clear whether most candidate molecules cross the placenta, signal through placental receptors, or influence foetal tissues through secondary mediators.

Longitudinal studies in humans are needed to determine whether maternal exercise is associated with changes in placental markers, childhood outcomes, and long-term cardiometabolic health across generations.

### 5.4. Proposed Role of Exerkines in Maternal–Placental–Foetal Communication

A conceptual framework linking exerkines with maternal–placental–foetal communication must account for several unresolved steps. Maternal exercise changes haemodynamics, substrate use, inflammatory activity, and circulating signalling molecules. The placenta can respond to these maternal changes and can release its own hormones, cytokines, and extracellular vesicles that influence maternal and fetal tissues [59].

Exerkines could act on placental receptors, alter trophoblast or endothelial function, or stimulate secondary placental signals. For most candidate molecules, however, placental transfer, tissue origin, receptor engagement, and concentration-response relationships during pregnancy have not been established. Improvements in maternal metabolism and blood flow may also affect the placenta without requiring direct exerkine action.

**Figure 1:**
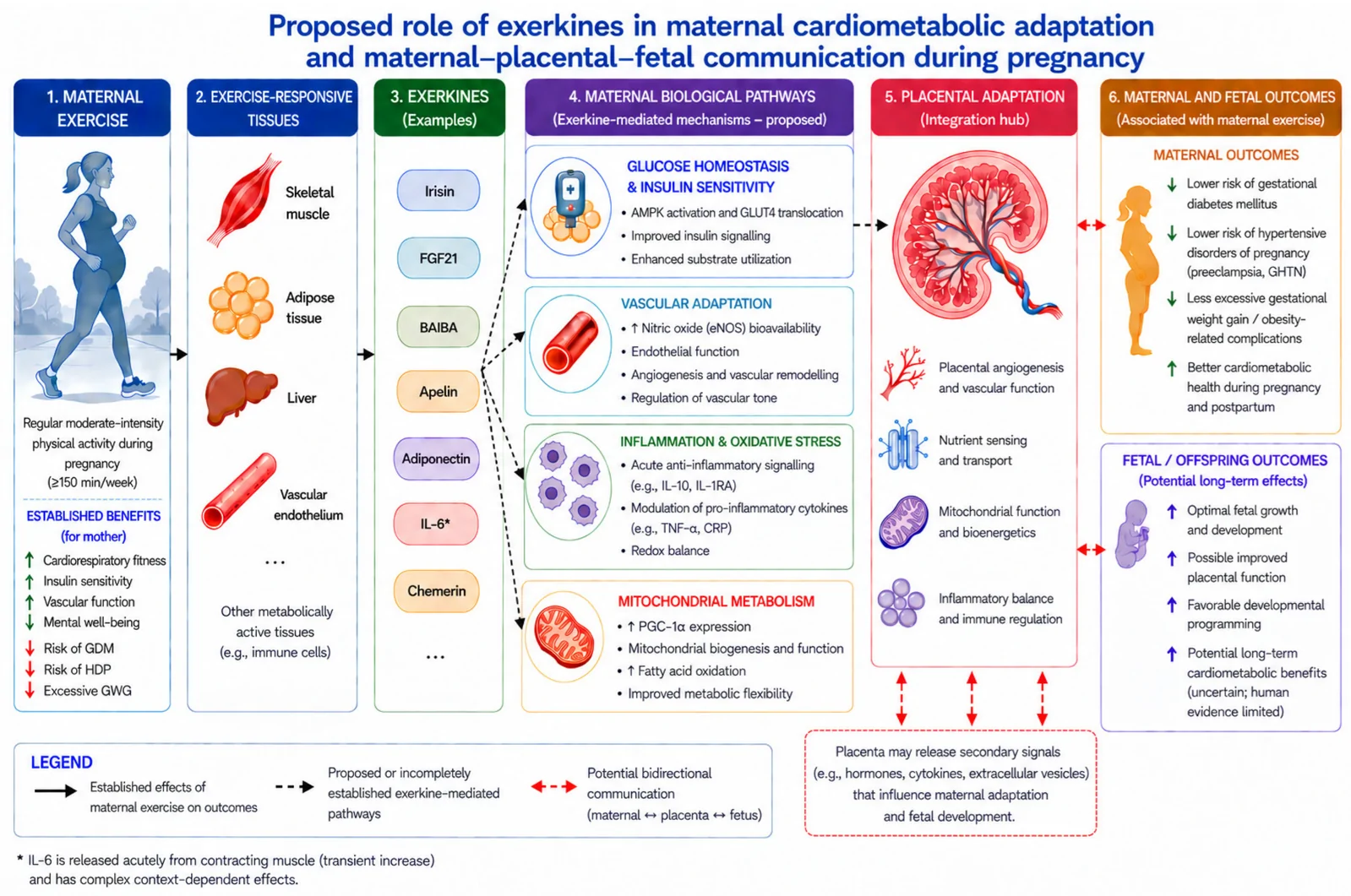
Conceptual framework for the potential role of exerkines in maternal cardiometabolic adaptation and maternal–placental–foetal communication during pregnancy. Maternal exercise is associated with changes in exercise-responsive factors (exerkines) from skeletal muscle and other tissues. These factors may contribute to improvements in glucose homeostasis, vascular function, inflammation, and mitochondrial metabolism, which may influence placental adaptation. The framework integrates established clinical effects of prenatal exercise with biological pathways and candidate exerkines for which the strength of evidence varies. The arrows represent proposed relationships and should not be interpreted as demonstrated causal pathways. Abbreviations: GDM, gestational diabetes mellitus; HDP, hypertensive disorders of pregnancy; GHTN, gestational hypertension; GWG, gestational weight gain; AMPK, adenosine monophosphate-activated protein kinase; FGF21, fibroblast growth factor 21; BAIBA, β-aminoisobutyric acid; eNOS, endothelial nitric oxide synthase; PGC-1α, peroxisome proliferator-activated receptor gamma coactivator 1-alpha.

Current evidence suggests that maternal exercise produces several parallel physiological changes, some of which may involve exerkines. These changes may influence placental angiogenesis, nutrient sensing, inflammatory balance, and cellular metabolism, with potential consequences for foetal exposure and development. No single molecule currently explains the broad effects of prenatal exercise. The conceptual integration of maternal exercise, exercise-responsive exerkines, maternal cardiometabolic pathways, placental adaptation, and maternal–placental–fetal communication is summarised in Figure 1.

Future studies should examine exercise exposure, maternal physiological responses, circulating exerkines, placental changes, and foetal or neonatal outcomes together within the same study. This would clarify if exerkines are actually involved in communication across the maternal–placental–foetal unit. Until then, this conceptual framework should be considered a clinically relevant hypothesis rather than an established mechanism.

## 6. Future Perspectives

Future research should focus on clinical translation rather than the continued description of isolated exercise-responsive molecules. At present, no individual exerkine has sufficient evidence to support its routine use for prediction, diagnosis, or monitoring of GDM, HDP, placental dysfunction, or foetal growth abnormalities.

Candidate exerkines may prove more informative when considered alongside established clinical characteristics and conventional metabolic or vascular markers. Such panels could eventually help identify women who respond differently to standardised exercise programmes, but this objective requires prospective validation. Interindividual variability in exerkine responses may be particularly relevant in this context. In non-pregnant populations, the same exercise stimulus can produce markedly different changes in circulating exerkines between individuals [15]. Whether similar responder and non-responder patterns occur during pregnancy remains unknown.

Current physical activity recommendations are appropriate for most uncomplicated pregnancies. More individualised programmes may be useful for women with obesity, previous GDM, chronic hypertension, or other cardiometabolic risk factors. Personalisation should initially be based on clinical assessment, safety, fitness, preferences, and adherence. If future biomarker-guided exercise prescription becomes feasible, molecular profiling could complement but not replace established clinical assessment and individualised exercise counselling.

Future trials should clearly describe exercise frequency, intensity, duration, volume, timing, and modality and should objectively assess adherence. Longitudinal sampling should include standardised gestational time points and, where feasible, measurements immediately before and after defined exercise sessions to distinguish acute exercise responses from chronic training adaptations and pregnancy-related changes. Studies should integrate maternal blood sampling with placental tissue and umbilical cord blood collected at delivery, together with maternal metabolic and vascular outcomes and neonatal measures. Multicentre studies would improve statistical power and generalisability. Long-term offspring follow-up is particularly important because most developmental programming evidence is still very limited [32].

Most clinical studies evaluating exerkines during pregnancy have measured circulating concentrations under basal conditions. Response to structured exercise interventions was rarely evaluated. This makes it difficult to distinguish pregnancy-related metabolic changes from true exercise-induced endocrine responses [18].

Another challenge in available research is the lack of consistency in how exerkines are measured across studies. Reported concentrations can be influenced by the type of biological sample used, particularly serum versus plasma, as well as by the timing of sample collection, gestational age, fasting status, and time of day. Exercise modality and the assessment of acute versus chronic responses may introduce further variation.

Maternal adiposity, baseline fitness, and metabolic status may further modify exerkine responses. These methodological differences complicate comparisons between studies. Greater methodological consistency would make findings across studies easier to compare. Sampling procedures and exercise protocols should be standardised, and analytical methods should be validated. Repeated measurements across pregnancy would also help determine if exerkine responses change with gestational age.

Multi-omics approaches could help move the field beyond measurement of isolated circulating molecules. Targeted and untargeted proteomic and metabolomic analyses of maternal blood and umbilical cord blood, combined with placental transcriptomic and proteomic profiling, could identify coordinated exercise-responsive pathways across the maternal–placental–foetal unit. Extracellular vesicle profiling may provide additional information on inter-tissue communication, although reliable attribution of circulating vesicles to skeletal muscle or other specific tissues remains technically challenging. Integration of these molecular data with standardised exercise exposure and clinical outcomes could help distinguish candidate mediators from biomarkers that merely reflect maternal metabolic status. Such approaches will require replication and validation in independent populations before clinical application. Exercise mimetics are still experimental and cannot reproduce the broad physical and psychological effects of maternal activity [8,14,15,114].

## 7. Strengths and Limitations

A strength of this narrative review is its integration of clinical evidence on prenatal exercise with pregnancy-specific and experimental evidence concerning exerkines, placental adaptation, and maternal–placental–foetal communication. Particular attention was given to distinguishing established clinical effects from pregnancy-specific associations and from mechanisms supported primarily by experimental or non-pregnant studies.

Several limitations should also be acknowledged. First, this is a narrative review based on a comprehensive, iterative literature search rather than a systematic review with a predefined screening protocol. Although multiple databases and reference lists were searched, the selection of literature was guided by relevance to the questions addressed, and some relevant publications may therefore not have been captured. Second, the evidence base is heterogeneous with respect to study populations, gestational age, exercise modality and exposure, sampling procedures, and analytical methods, which limits direct comparison across studies. Pregnancy-specific evidence is also uneven across candidate exerkines, and for several molecules the proposed biological mechanisms rely substantially on experimental or non-pregnant research. Finally, circulating exerkine concentrations do not establish tissue origin or causal mediation. This is particularly relevant during pregnancy, when skeletal muscle, adipose tissue, the placenta, and other metabolically active tissues may contribute to the circulating pool of exercise-responsive signalling molecules. These limitations preclude firm conclusions regarding the causal contribution or clinical utility of individual exerkines during pregnancy.

## 8. Conclusions

Regular physical activity is an established component of antenatal care and reduces the risk of GDM, HDP, and excessive gestational weight gain while supporting maternal fitness and psychological well-being. These clinical effects are supported more strongly than any proposed molecular explanation.

Exerkines provide a credible framework for understanding how maternal exercise may influence multiple physiological systems during pregnancy. Pregnancy-specific evidence remains strongest for irisin, although published findings are heterogeneous. Acute exercise studies have also demonstrated transient increases in circulating FGF21, whereas evidence for BAIBA, apelin, adiponectin, chemerin, and other candidate exerkines derives predominantly from experimental models or non-pregnant populations. Consequently, the physiological and clinical significance of most exercise-induced exerkine responses during pregnancy remains to be established.

The placenta is likely to play a central integrative role in translating maternal physiological adaptations into foetal developmental signals. Direct exerkine-mediated communication across the maternal–placental–foetal unit has not yet been demonstrated in humans, and long-term offspring benefits remain more convincingly supported by animal than human research.

Exerkines should therefore not currently be used as clinical biomarkers or therapeutic targets in pregnancy. Their main value is as research candidates that may help explain the broad effects of exercise and identify variation in physiological response. Carefully designed longitudinal trials that integrate clinical outcomes with repeated biomarker and placental measurements are needed.

The practical conclusion remains clear: appropriately prescribed exercise benefits pregnancy. Better scientific evidence may eventually refine exercise prescription, but it should strengthen rather than delay the implementation of current clinical recommendations.

## Data Availability

No new data were created or analysed in this study. Data sharing is not applicable to this article.

## Abbreviations

The following abbreviations are used in this manuscript:

ACOG	American College of Obstetricians and Gynecologists
AdipoR1	adiponectin receptor 1
AdipoR2	adiponectin receptor 2
AKT	protein kinase B
AMPK	adenosine monophosphate-activated protein kinase
APJ	apelin receptor
BAIBA	β-aminoisobutyric acid
CMKLR1	chemokine-like receptor 1
CSEP	Canadian Society for Exercise Physiology
DOHaD	Developmental Origins of Health and Disease
eNOS	endothelial nitric oxide synthase
FGF21	fibroblast growth factor 21
FNDC5	fibronectin type III domain-containing protein 5
GDM	gestational diabetes mellitus
GHTN	gestational hypertension
GLUT3	glucose transporter type 3
GLUT4	glucose transporter type 4
GWG	gestational weight gain
HDP	hypertensive disorders of pregnancy
IL-1	interleukin-1
IL-6	interleukin-6
IL-6R	interleukin-6 receptor
IL-10	interleukin-10
ISSHP	International Society for the Study of Hypertension in Pregnancy
MAPK	mitogen-activated protein kinase
mTOR	mechanistic target of rapamycin
PGC-1α	peroxisome proliferator-activated receptor gamma coactivator 1-alpha
PI3K	phosphatidylinositol 3-kinase
PlGF	placental growth factor
PPAR-α	peroxisome proliferator-activated receptor-α
ROS	reactive oxygen species
sEng	soluble endoglin
sFlt-1	soluble Fms-like tyrosine kinase-1
STAT4	signal transducer and activator of transcription 4
TNF-α	tumour necrosis factor-α
VEGF	vascular endothelial growth factor
VEGFR-1	vascular endothelial growth factor receptor-1
WHO	World Health Organisation
